# Evaluation of a genome-wide approach to multiple marker association considering different marker densities

**DOI:** 10.1186/1753-6561-3-s1-s5

**Published:** 2009-02-23

**Authors:** Matthew A Cleveland, Nader Deeb

**Affiliations:** 1 Genus plc., 100 Bluegrass Commons Blvd., Suite 2200, Hendersonville, TN, 37075, USA

## Abstract

**Background:**

Genome-wide approaches to analyze single nucleotide polymorphism (SNP) data have proliferated due to the increased availability and affordability of markers, but in practice a small number of markers may be selected from sets that do not approach dense genome-wide coverage. This study focused on a genome-wide approach to identify markers useful to a breeding program using a Bayesian method to estimate effects for markers distributed across the genome at varied densities. A simulated dataset containing 4665 individual phenotypes for a quantitative trait and genotypes for 6000 SNPs spaced in 0.1 cM increments across six chromosomes was analyzed using a Bayesian approach in which effects for all single markers are simultaneously estimated. The dataset was also analyzed with marker densities reduced to 0.5, 1.0, 2.0 and 5.0 cM. Type I errors were not a major concern but replications of each analysis were performed to determine acceptance of estimated marker effects.

**Results:**

The Bayesian analysis of the original dataset was able to estimate genetic values for markers in a small number of regions while shrinking other marker effects to zero. Analysis of the reduced density datasets also showed clear signals in a small number of regions where some effects appeared to be distributed across multiple markers. Replicates of the analyses provided evidence for regions with moderate and large effects.

**Conclusion:**

A Bayesian multiple marker approach appears to be suitable for predicting genetic values, even with reduced density datasets where large numbers of markers are not yet available for many species. These predicted genetic values can be implemented in marker assisted selection programs.

## Background

Genome-wide approaches to analyze single nucleotide polymorphism (SNP) data have proliferated due to the increased availability and affordability of large-scale genotyping efforts. Meuwissen et al. [1] introduced Bayesian methods to predict genetic values of animals for selection using SNP haplotypes with dense genome-wide coverage. Xu [2] applied this approach to both real and simulated populations in a quantitative trait loci (QTL) mapping framework, using genome-wide SNPs. These methods generally assume marker density is high and animals can be genotyped at each stage for all markers, which may not always be realistic. In practice only a relatively small number of SNPs may be included in a genetic evaluation system and the set from which they are selected may not approach dense genome-wide coverage, for example, in pigs a complete genome map and high density chips are not currently available. Even with these limitations genome-wide strategies may be used to identify the best subset of SNPs to use in marker assisted selection (MAS), but little is known about how these strategies perform when density is low by necessity, compared to more optimal levels.

This study used a Bayesian method originally designed for genome-wide selection to estimate effects for SNPs distributed across the genome and evaluated the behaviour of a multiple marker approach to identify interesting marker subsets and genomic regions that may be useful for MAS programs, when densities were varied.

## Methods

The dataset for analysis was simulated as part of the 12^th ^QTLMAS Workshop, see [3] for details. A subset of the data containing 4665 individual phenotypes for a quantitative trait and genotypes for 6000 SNPs spaced in 0.1 cM increments across six chromosomes was used in the analysis. The first SNP on each chromosome was positioned at zero cM and subsequent SNPs assigned relative positions. Minor allele frequencies (MAF) were calculated and SNPs with MAF < 0.001 were excluded, yielding 5923 SNPs from the original set. The exclusions meant that the distance between some SNPs was greater than 0.1 cM, but the increase in overall average distance was very small (from 0.1 to 0.101 cM).

Marker (QTL) effects were estimated using the Bayesian procedure described by Meuwissen et al. [1] and Xu [2] where all single markers are fit simultaneously as random variables to estimate posterior means and variances for each. The terms marker and QTL are used interchangeably throughout, where a marker is the QTL or is representative of a QTL in proportion to the linkage disequilibrium (LD) between the two. In this case the marker effect included the additive component only. The prior for the marker variance followed ter Braak et al. [4] where the variance is sampled from an inverse chi-square distribution with (1–2δ) d.f.; δ = 0.002 for this study. A Bayesian multiple-marker analysis (MMA) was performed separately for each chromosome, due to the computational resources required, under the assumption that LD across chromosomes was minimal. Each analysis included 50,000 Gibbs samples where the first 20,000 sampled parameter values were discarded as burn-in. Following burn-in, samples were saved for every 20 rounds. Posterior means are reported as marker (additive) effects.

A single marker analysis (SMA) using a standard BLUP approach was also performed on all SNPs. Each SMA included the additive effect of a single marker as a fixed factor and the effect of pedigree (a random animal effect) so the analysis could be used as a comparison to the Bayesian approach where the effect of pedigree should be implicitly included with dense marker coverage.

Each analysis was performed on the original data subset, using all phenotypes, where marker spacing was 0.1 cM and additionally with marker spacing set at 0.5, 1.0, 2.0 and 5.0 cM. The new subsets contained (with low MAF markers removed) 1179, 588, 296 and 116 markers, respectively. Type I errors were not a major concern as the specific location of QTL are not necessarily of interest when identifying markers for breeding programs, but analyses were replicated 100 times as a measure of the reliability of the estimated marker effects and, thus, the usefulness of the markers.

## Results

### Computation time

All Bayesian analyses were performed on a standard quad-core Windows PC (Intel Xeon 3.00 GHz processor; 3.00 GB RAM) implemented in a serial Fortran program. Simple modifications were made to the published algorithm [2] to improve computational efficiency. The time needed to run the full dataset (0.1 cM spacing) was ~11 h per chromosome, dropping considerably with the reduction in number of markers. At 0.5 cM spacing the runtime was 60 min per chromosome, 33 min at 1.0 cM, 7 min at 2.0 cM and 2 min at 5.0 cM.

### Original dataset

**Figure 1:**
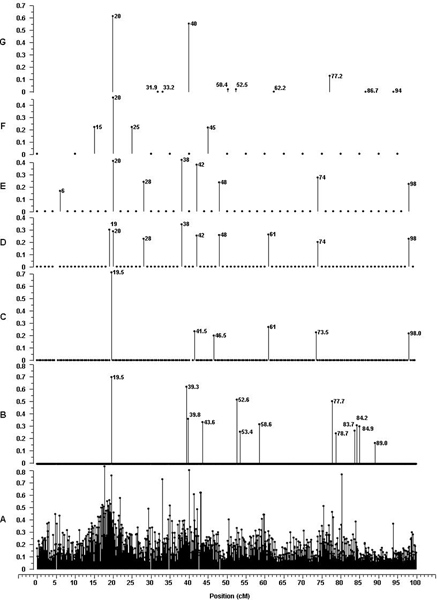
**Marker effects plotted by genome location for chromosome 1**. (A) Single marker regression using all markers; (B) Bayesian multiple marker analysis with marker spacing of 0.1 cM, (C) 0.5 cM, (D) 1.0 cM, (E) 2.0 cM and (F) 5.0 cM; (G) Simulated QTL (see [3]). The vertical axis is the absolute marker (QTL) effect. The labels are the position of markers with non-zero effects in the Bayesian analysis, or the position of the simulated QTL.

The marker effects estimated by the Bayesian MMA and SMA are shown in Figure 1, plotted against genome location. Results are shown for chromosome 1 only for simplicity. The Bayesian analysis showed clear signals for 13 markers in 8 small regions across chromosome 1, whereas the rest of the marker effects were shrunk to zero. The number of marker peaks on the other chromosomes ranged from nine to sixteen with the average across all being nearly thirteen (results not shown). The single marker analysis yielded signals that were comparatively much less clear, though several regions with peaks of large effect indicate the presence of putative QTL.

In most cases the effects for single markers, or small marker groupings, identified by the Bayesian analysis corresponded with effects for single markers or small regions in the regression analysis in terms of position and effect size. In cases where there was not one-to-one correspondence between single marker peaks in the two analyses the multiple marker approach appears to distribute effects across multiple nearby markers in a given region marking a presumed QTL. This is evidenced by the fact that some marker peaks in the Bayesian analysis (e.g., at 19.5 cM) closely match the magnitude of effect in the single marker analysis while other peaks (e.g, at 39.3 and 39.8 cM) appear to combine to represent a single QTL effect.

One peak in the Bayesian analysis (at 89 cM) represented the smallest of the estimated effects and was not identified by the SMA. In Bayesian terms there is only a small amount evidence for a QTL at this location and because the Bayesian approach does not generally consider significance of effects, this peak is simply identified as having a small posterior estimate in a single run. More evidence of an effect (or lack of effect) was obtained from evaluation of the dataset replication (see below).

The use of the replicate analyses provides some measure of the reliability of, or support for, marker effect estimates (results not shown). The replicates showed that the peaks at 19.5 and 77.7 cM (Figure 1), near a large and moderate QTL respectively (see [3]), were represented in at least one-half of the replicates. Peaks at 39.3, 39.8, 52.6 and 53.4 cM were near (<2 cM) simulated QTL and also represented small regions appearing in multiple replicates, though less than 50 percent of the time in all cases. Peaks at 58.6, 83.7 and 89.0 cM were more distant from QTL, where the first peak had little support from the replicates, but the second and third appeared more than 20 times and were apparently false positive results.

### Datasets with varied marker densities

The marker effects estimated by the Bayesian MMA for marker spacing 0.5, 1.0, 2.0 and 5.0 cM are shown in Figure 1, plotted against genome location. Results are presented for chromosome 1 only. The Bayesian analysis shows clear signals for effects when markers were spaced from 0.5 to 5.0 cM apart. The number of peaks was similar across all chromosomes but the average number decreased from nearly eight at the 0.5 cM spacing to two and one-half at 5.0 cM (results not shown). The locations of the peaks on chromosome 1 compare well with those in the original dataset, where the magnitude of the absolute effects was similar. The largest peak in the Bayesian analysis of the original dataset was at 19.5 cm (near the largest QTL at 20.0 cM [3]), which is seen also in the 0.5 cM spacing dataset. With the increase in spacing to 1.0 cM the peak at 19.5 cM disappears as this marker is no longer included in the analysis, but the effect is divided between the two closest flanking markers where the sum of the absolute effects nears the magnitude of the original peak. Analysis with the 2.0 and 5.0 cM datasets also shows a peak near the 19.5 cM position with smaller effects sizes than the original peak, but larger than the effect of the same marker in the 1.0 cM dataset, where part of the effect was apparently absorbed by the flanking marker.

Fewer peaks were observed with the reduced densities, compared to the original, as might be expected where the probability of a marker being in LD with a QTL is reduced with increased distances. There is seemingly also less likelihood of multiple markers sharing QTL effects causing multiple peaks. The combination of reduced LD and small effect sizes reduces the likelihood of sampling non-zero effects, which may or may not be real, and could explain the disappearance of the peak at 89 cM in the original analysis and why the effect was not shifted to a nearby marker. Conversely, the addition of new peaks from analysis of the reduced density datasets was unexpected. A peak between 73 and 74 cM in the 0.5, 1.0 and 2.0 analyses is distant from any previously identified peaks, including in the single marker regression. It is possible that the reduction in marker density, which results in certain markers being removed from the analysis, shifts the effect from a more distant QTL (e.g., the original peaks ~77 cM representing a real QTL, but not present in the reduced analyses) and that this new peak actually represents this QTL effect. There was no support for a QTL effect between 73 and 74 cM in the analysis replicates and indeed a QTL was not simulated in this region [3]. A similar situation was observed for a peak at 98 cM (0.5, 1.0 and 2.0 cM spacing), which was not near a simulated QTL. More work is needed using real data to evaluate such effects.

Interestingly, even with markers spaced at 5 cM, a distance where average LD between adjacent markers, and presumably between markers and QTL, will be low in outbred livestock species, two peaks on chromosome 1 consisting of four markers were identified by the Bayesian analysis. These peaks correspond with peaks in all of the previous analyses, including the single marker regression, and were suggestive of large QTL effects in these regions. In fact, each of the four markers appeared in nearly 100 percent of the replicates for this dataset (5.0 cM spacing). The peaks at 15, 20 and 25 cM are the nearest markers to the large QTL simulated at 19.5 cM and the peak at 45 cM was the nearest marker (except the marker at 35 cM, which was equidistant) to the large QTL at 40.0 cM [3]. Curiously, the marker located at 40.0 cM had effects shrunk to zero in all analyses. Genomic regions with smaller QTL were not identified when density was reduced to 5 cM.

## Discussion

Xu [2] applied the Bayesian method described by Meuwissen et al. [1] to real and simulated populations to estimate polygenic effects using genome-wide SNPs, with marker densities much lower than in the original work (5 and 11 cM). Xu found clear signals for putative QTL that corresponded very closely to the large peaks in a single marker regression, for the real data, and were at the position of the true QTL in the simulated data. Effects that were large in the single marker regression were large in the Bayesian analysis, while smaller effects were shrunk. In the current study there were multiple peaks in close proximity that were identified with dense marker coverage (0.1 cM spacing) and the regions with larger effects in the single marker analysis were generally identified by the Bayesian MMA, where effect sizes were of similar magnitude. The marker densities were greater in the current study for all datasets, except one, and it is likely that the close proximity of the markers resulted in a distribution of effects among nearby markers and thus multiple peaks in the same region. Replications of the Bayesian analysis highlight this effect as regions with small QTL yielded non-zero marker effects in multiple positions, while regions with large QTL were identified at few positions in many replicates. The Bayesian approach using dense coverage, however, was able to isolate QTL effects, even if distributed across multiple markers, and shrink many spurious effects to zero. The replicate analysis was also able to identify peaks (or small regions) where there was little support for a QTL in that region. In the original dataset a replicate threshold of 50 percent would yield two markers near two of the largest QTL, but would discard several markers in other interesting regions including the markers near the large QTL at 40.0 cM, along with regions not containing a QTL. Further work is needed to identify the appropriate replicate size and threshold level, especially when density is high.

Results from this study showed a decrease in the number of effect peaks with a reduction in marker density, but major regions (e.g. particularly around the QTL at 19.5 and 40.0 cM) were still clearly identified by nearby markers when the closest markers to a particular putative QTL were no longer in the dataset. There is a dearth of literature detailing the effect of decreased density on genome-wide selection approaches for marker association or QTL mapping; however this work describes a potential intermediate step in the calculation of genomic breeding values when high density coverage is not practical (or possible). Solberg et al. [5] found a decrease in accuracy of estimated breeding values (EBV) calculated from single marker effects (using a Bayesian approach) at a density of 1 cM versus 0.5 cM, but still high (0.66) when considering marker information alone was used. Similarly, Calus et al. [6] found moderately high accuracies for EBV from marker effects predicted using a Bayesian model (but also including a polygenic effect) when average marker density was 2.59 cM. These results indicate that there is value in predicting genetic effects across the genome at lower densities, where even the two peaks identified on chromosome 1 in the current study, when the density was 5 cM, will add information to improve the accuracy of EBV. The marker effects that remain when density is reduced represent larger QTL that will account for most of the genetic variance and the smaller effects that might be missed using the Bayesian framework described here may have little impact on the total genetic value for a trait.

## Epilogue

The performance of the Bayesian method described here can be evaluated in light of the simulated QTL effects described in [3], with specific comparisons discussed previously in the text. The Bayesian multiple marker analysis was successful in identifying markers near the three QTL (on Chromosome 1) accounting for more than one percent of phenotypic variance and markers near one of the small QTL (52.5 cM), but effects were generally overestimated. Markers for the QTL at 50.4 cM were apparently missed, but the proximity of this QTL to the one at 52.5 cM may have caused some of the effects to be absorbed into markers nearer to 52.5 cM than 50.4 cM. In this case both QTL effects would be inherently captured by the model due to the simultaneous fit of the marker effects. There were four marker peaks that were apparent false-positives as they were distant from any simulated QTL (see Figure 1). A grouping of these peaks around 84 cM (identified in multiple replications) would suggest a nearby QTL, maybe absorbing some of the effect from the QTL at 77.2 cM, but the distance is seemingly too large for this to be the case. The reduction in density yielded different subsets of markers with non-zero effects, as the effects shifted, but markers near the two largest QTL (20.0 and 40.0 cM) were identified at each level. In general the Bayesian approach identified markers near QTL that are large enough to have practical implications for a breeding program, even when marker density was reduced, while the effect of identifying apparent false-positives and in overestimating effect sizes is not known.

## Conclusion

A Bayesian multiple marker approach was able to identify markers or small regions with effects corresponding to putative QTL with much clearer signals than in a single marker regression. Replication of the analyses provided some measure of acceptance for each of the marker effects. The Bayesian analysis performed well when marker density was reduced in part by distributing effects across adjacent markers. This approach appears to be suitable for predicting genetic values, even with reduced marker densities, where large numbers of markers are not yet available for many species. These genetic values can be implemented in MAS programs.

## Competing interests

The authors declare that they have no competing interests.

## Authors' contributions

MAC conceived the study, participated in its design, performed the analysis and drafted the manuscript. ND participated in the design of the study, evaluated the results and helped to draft the manuscript.
